# Real World Experience of Disease Activity in Patients With Rheumatoid Arthritis and Response to Treatment With Varios Biologic DMARDs

**DOI:** 10.3389/fphar.2018.01303

**Published:** 2018-11-20

**Authors:** Vladimira Boyadzhieva, Nikolay Stoilov, Mariana Ivanova, Guenka Petrova, Rumen Stoilov

**Affiliations:** ^1^Faculty of Medicines, Medical University of Sofia – University Hospital “St. Ivan Rilski”, Sofia, Bulgaria; ^2^Faculty of Pharmacy, Medical University of Sofia, Sofia, Bulgaria

**Keywords:** rheumatoid arthritis, disease activity, biological therapy, DAS28-CRP, CDAI

## Abstract

The current study investigate the disease activity and effectiveness of treatment in patients with RA on biological disease modifying antirheumatic drugs (bDMARDs) in combination with a conventional synthetic DMARD (csDMARD) and determine whether or not the benefits of different therapies were sustained over a follow up period of 1 year. 124 patients were selected with a mean age 55.26 ± 13, 18SD years, meeting the 1987 ACR and /or ACR/ EULAR (2010) classification criteria for Rheumatoid arthritis (RA). Patients were arranged according to treatment regimens: Tocilizumab (TCL) – 30 patients, Certolizumab (CZP) – 16, Golimumab (GOL) – 22, Etanercept (ETN) 20, Adalimumab (ADA) 20, Rituximab (RTX) – 16. Disease activities was the primary concern. Independent joint assessor evaluated 28 joints on baseline, 6th and 12th month’s thereafter. C-reactive protein (CRP) was used to measure the inflammatory process. DAS28-CRP, clinical disease activity index (CDAI) and simplified disease activity index (SDAI) were calculated. On baseline all of the patients’ groups had severe disease activity (mean DAS28-CRP > 5.2, mean CDAI > 22, mean SDAI > 26. It was noted that, during the 6th month follow-up period all of the treatment groups significantly decreased DAS28-CRP, CDAI, SDAI and reach moderate disease activity. After 6th and 12th months of treatment all of the groups on bDMARDs had significantly lower disease activity. The GOL group reach remission only according to DAS28-CRP: 2.49 ± 0.76, and low disease activity as measured by CDAI: 6.78 ± 4.51 and SDAI 7.80 ± 5.67. The other 5 groups after 12 months reach the level of low disease activity according to the three activity parameters: DAS28-CRP (TCL 3.07 ± 0.73, CZP 3.06 ± 0.65, ETN 2.85 ± 0.55, ADA 3.15 ± 0.82, RTX 2.90 ± 0.70), CDAI (TCL 9.80 ± 4.91, CZP – 9.33 ± 4.22, ETN 7.97 ± 3.80, ADA 10.00 ± 5.25, RTX 7.48 ± 2.99) and SDAI (TCL 10.45 ± 5.14, CZP 9.94 ± 4.43, ETN 9.03 ± 4.25, ADA 10.50 ± 5.61, RTX 8.08 ± 3.24). The therapy with different bDMARDs added to a csDMARD led to very similar results – a minimal disease activity and a state of remission in the GOL treatment group only as per DAS28-CRP.

## Introduction

Rheumatoid arthritis (RA) is a chronic, autoimmune inflammatory disorder characterized by erosion-destructive progressive symmetrical arthritis. The disease is likely to affect not only the joints but also to have systemic impairments as well. The causes of RA are not yet sufficiently known, but the genetic predisposition is a major factor which acts in conjunction with additional factors such as epigenetic modifications and environmental factors (microbiomes, infections and drug, and toxin-exposure) (Deane et al., 2017). RA affects about 0.5% of the population (Woolf and Pfleger, 2003). According to the European Federation of Pharmaceutical Industries and Associations (EFPIA) report in 2008, 29,711 (0.4%) people with RA suffered from it (Kobelt and Kasteng, 2009).

Rheumatoid arthritis evolves relatively slowly but progressively, and usually periods of aggravation and remission alternate with each other. Each attack results in rapid joint damage, deterioration of the functional capacity of the musculoskeletal system and to a different degree of disability. Measurement of the disease activity is one of the main considerations in the choice of a therapeutic approach to prevent the disability of these patients (van der Heijde et al., 1990; Neogi and Felson, 2008; Ringold SaS, 2008; Taylor and Bagga, 2011).

After a detailed analysis of the methodologies in 2012, the ACR recommends six of them, which measure a single index and define the categories of low, moderate and high disease activity or clinical remission. The use of the six methodologies: DAS28, CDAI (clinical disease activity index), SDAI (simplified disease activity index), PASS, PASS II, RAPID-3, has proven their relevance in the disease monitoring in an everyday clinical practice (Felson et al., 1993; Anderson et al., 2012).

The disease-modifying antirheumatic drugs (DMARDS) affect the natural course of the disease, and in most cases sustained suppression of its inflammatory activity (Smolen et al., 2013). In the absence of an adequate therapeutic response to conventional synthetic DMARDS (csDMARDS) in combination with or without corticosteroids and non-steroidal anti-inflammatory drugs (NSAIDs), treatment with biological disease-modifying antirheumatic drugs (bDMARDS) should be initiated according to the updated EULAR recommendations for the management of RA developed in 2016 (Smolen et al., 2016).

The aim of the treatment for RA is remission. Maintaining minimal disease activity is an alternative goal in patients who cannot achieve lasting remission especially in those with long-standing disease (Felson et al., 2011). When there is good control of disease activity, the ability to work improves, thereby increasing labor productivity. In recent years, the improvement of this indicator is mainly due to the introduction of more effective therapies (Felson et al., 1993). The use of bDMARDs for RA therapy is an extremely important treatment approach that has reduced the disability of patients in recent years (Strand et al., 2016).

There are numerous reasons that have made our study necessary: the growing importance of biological treatment over the past 10 years, the growing number of biological medicinal products on the market, the data from many other population clinical studies in this field, and the lack of such data among the Bulgarian population.

The main objective is to evaluate the disease activity and the response to treatment with biological DMARDs in RA patients from Bulgaria for the period from 2012 to 2016.

## Materials and Methods

This is a prospective, follow up, real life study of 124 patients treated with biological agents in combination with a csDMARD in a dosing which have been kept stable before and throughout the study during 2012–2016 at the University hospital “St. Ivan Riskli” in Sofia, Clinic of rheumatology. The initial number of selected patients was 143, but 19 patients were excluded due to a switch between therapies and failure to comply with the inclusion criteria.

Patients meeting the criteria of the study protocol, which was approved by the Ethics committee of the institution were included in the prospective analyzes. All participants gave informed consent in accordance with the Declaration of Helsinki.

Inclusion criteria were: age above 18 years; willingness to participate after informed consent; confirmed diagnosis of RA according ACR (1987) and / or ACR/EULAR (2010) (Aletaha et al., 2010); treatment naïve on biological therapy; previous treatment with methotrexate and nonsteroidal anti-inflammatory drugs or methotrexate and other disease modifying therapy; adherence to therapy in the previous 6 months and during the whole period of observation.

Exclusion criteria were infectious diseases (HIV, tuberculosis); cardiac insufficiency (NYHA III and IV grade); malignant hypertension; psychiatric diseases; any neoplasms or proliferative lymph diseases within the previous 5 years (Dolgin et al., 1994); alcohol or narcotic abuse; deficiencies in recognition abilities.

According to the regulations of the National Health Insurance Fund (NHIF), patients with intolerance of two or more csDMARDS, can start therapy with biological agents. In our study we have selected only patients on a stable treatment with csDMARD for at least 6 months to standardize the results obtained for disease activity. The inclusion criteria of NHIF are:

(1) Diagnosis of RA (ACR criteria 1987).
(2) Age > 18 years.
(3) Non-responders to conventional synthetic DMARDs at optimal doses for 6 months – therapy should have failed at least two of them (one of which must be Methotrexate 20 mg/weekly, and one from the following – Arava 20 mg/daily, Resochine 250 mg/daily or Salazopirine 3 g/daily).
(4) Disease activity score (DAS28, ESR or CRP) > 5.1.
(5) If patients are intolerant to Methotrexate monotherapy, or other csDMARD they can start bDMARD as a monotherapy.

The exclusion criteria of the NHIF are the same as selected for the study.

In total, 110 female and 14 male were selected. Rheumatologist chose the biological medicines according to their personal opinion based on the corresponding clinical status of the patients and available drugs in the reimbursement list (as for Rituximab, this met the requirement as a second-line therapy option after failure of one TNF inhibitor). All of the patients were on stable therapy according to the inclusion criteria, and didn’t interrupt any of the medications including biological treatment. Adherence to the therapy was confirmed with patient anamnesis on each visit, patient prescription book (the date of each application can be check) and pharmacy stamp and date on protocol for biological treatment issued by NHIF.

On the basis of the rheumatologist’s choice the patients were allocated to six treatments as followed: tocilizumab (*n* = 30), certrolizumab (*n* = 16), golimumab (*n* = 22), etanercept (*n* = 20), adalimumab (*n* = 20), rituximab (*n* = 16), administered in the approved doses.

Independent joint assessor evaluated 28 joints on baseline, at the 6th and 12th month of follow-up period. C-reactive protein (CRP) was used as an inflammatory biomarker to measure the inflammatory process. The disease activity state was evaluated by calculation of the disease activity score (DAS28-CRP), CDAI and SDAI according to the standard formulas. As CRP is now widely accessible, and is responsive in a timelier manner to changes in systemic inflammatory activity, the DAS28—CRP is widely used today, which is the main reason to select it for the study (Fransen et al., 2003; Carl et al., 2018).

Patient assessment of disease related pain, global health and physician global assessment of disease activity (VAS – 100 mm) were measured in order to estimate CDAI, SDAI, DAS28-CRP. The patient’s disease activity was measured every 6th month of treatment. Also, patients were assessed over the same interval whether they have reached a sufficient therapeutic response (DAS28 reduction of 1.2) to ongoing biological treatment or have to be switched to another bDMARD due to lack or insufficient effectiveness.

The data was analyzed using SPSS version 13 (SPSS Inc., Chicago, IL, United States). Means with SDs and percentages were calculated to ascertain demographic and clinical characteristics of the study subjects. Then comparisons between study groups were carried out using the *t*-test – Tamhane’s conservative pairwise comparisons test – that do not assume equal variances and LSD to perform all pairwise comparisons between group means. No adjustment was made to the error rate for multiple comparisons. Comparisons were also performed by the analysis variance ANOVA. The 2-tailed *p-*values of < 0.05 were considered to be statistically significant.

The basic demographic characteristics of the patients included in the study are shown in Table 1. No substantial differences were found between the patient’s groups treated differently.

**Table 1:** Demographic characteristics and treatment regimens of the selected patients.

Demographic characteristics:	Tocilizumab	Certolizumab-pegol	Golimumab	Etanercept	Adalimumab	Rituximab	All patients on bDMARDS
Number of patients(%)	30 (24%)	16 (13%)	22 (18%)	20 (16%)	20 (16%)	16 (13%)	124 (100%)
Age(years), mean ±*SD* (range)	55.1 ± 13.28	58.68 ± 13.35	53.77 ± 12.18	56.15 ± 12.58	53.2 ± 12.37	55.69 ± 13.22	55.26 ± 13.38
Gender (%)	3% male 97% female	3% male 97% female	9% male 91% female	20% male 80% female	15% male 85% female	19% male 81% female	11% male 89% female
Disease duration (years), mean ±*SD*	14.13 ± 10.6	9.38 ± 10.49	10.81 ± 9.33	11.5 ± 9.18	12.8 ± 8.8	9.32 ± 8.85	11.67 ± 8.43
RF(+)positivity, *n*(%)	21 (70%)	15 (94%)	17 (77%)	18 (90%)	17 (85%)	15 (94%)	103 (83%)
Anti-CPP(+)positivity, *n* (%)	15 (50%)	12 (75%)	12 (55%)	13 (65%)	14 (70%)	11 (69%)	77 (62%)


## Results

### Evaluation of Disease Activity (DAS28-CRP, CDAI, and CDAI)

**FIGURE 1:**
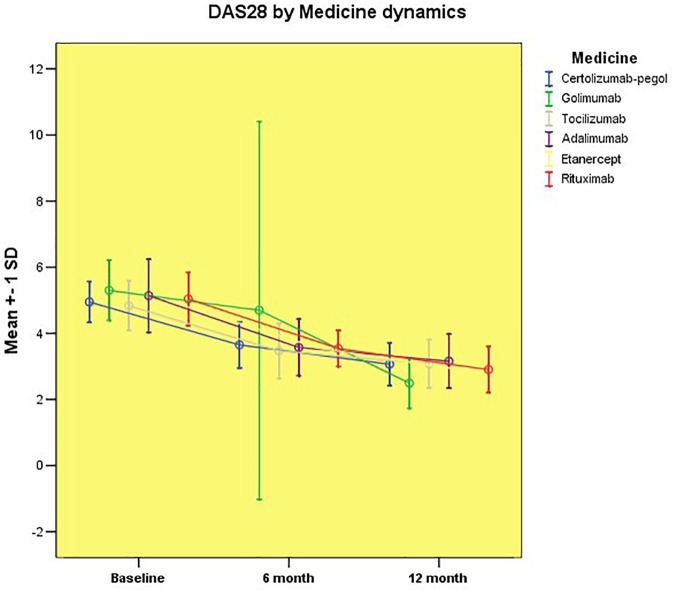
Disease activity assessed by DAS28-CRP by groups.

We analyzed the changes from the baseline in DAS28-CRP, CDAI and SDAI at 6 and 12 month intervals to evaluate the response to the treatment. The comparisons showed that all the treatment groups achieved a significant reduction in all of the disease activity parameters at both assessment time periods (Figure 1 and Table 2).

**Table 2:** Mean values of disease activity by groups (*p* < 0.05).

bDMARDS	DAS28-CRP (mean ± SD) Baseline	CDAI (mean ± SD) Baseline	SDAI (mean ± SD) Baseline	DAS28-CRP (mean ± SD) 6th month	CDAI (mean ± SD) 6th month	SDAI (mean ± SD) 6th month	DAS28-CRP (mean ± SD) 12th month	CDAI (mean ± SD) 12th month	SDAI (mean ± SD) 12th month
									
Certolizumab pegol	4.95 ± 0.74	25.1 ± 6.42	27.44 ± 7.4	3.65 ± 0.69	13.73 ± 5.3	14.82 ± 5.35	3.06 ± 0.65	9.33 ± 4.22	9.94 ± 4.43
Golimumab	5.3 ± 0.70	27.6 ± 6.2	31.2 ± 7.1	4.69 ± 5.71	13.41 ± 4.3	14.73 ± 5.43	2.49 ± 0.76	6.78 ± 4.51	7.80 ± 5.67
Tocilizumab	4.82 ± 0.75	22.7 ± 1.63	25.96 ± 9.45	3.47 ± 0.83	14.2 ± 4.99	15.37 ± 5.41	3.07 ± 0.73	9.80 ± 4.91	10.45 ± 5.14
Adalimumab	5.14 ± 0.78	26.7 ± 7.15	29.94 ± 8.33	3.57 ± 0.85	13.58 ± 6.45	14.80 ± 6.57	3.15 ± 0.82	10.00 ± 5.25	10.50 ± 5.61
Etanercept	4.98 ± 0.79	24.3 ± 7.45	27.7 ± 8.6	3.55 ± 0.49	12.38 ± 4.24	13.82 ± 4.96	2.85 ± 0.55	7.97 ± 3.80	9.03 ± 4.25
Rituximab	5.04 ± 0.84	24.9 ± 7.55	27.99 ± 8.86	3.54 ± 0.54	12.83 ± 471	8.08 ± 3.24	2.90 ± 0.70	7.48 ± 2.99	8.08 ± 3.24


After 6 months of treatment, we observed a marked reduction in the level of disease activity in all of the treatment groups, changing from a high to a moderate activity state (evaluated by the three instruments). With longer follow-up periods, we found that the mean values of rheumatoid inflammation in all of the groups significantly decreased to a level of low /minimal activity. It is noteworthy, that only the Golimumab treatment group reached the remission category according to DAS28-CRP, but compared to the other two CDAI and SDAI methodologies, the results continue to be categorized as low disease activity. Figure 1 demonstrates the change in disease activity between the mean values of patients of different treatment regimens as assessed by the DAS28-CRP score.

There was no statistically significant difference between the mean values of DAS28-CRP regardless of treatment with either Certolizumab pegol, Golimumab, Tocilizumab, Adalimumab, Etanercept, or Rituximab. The achieved therapeutic results for this observational period showed similar efficacy of the biological treatment in the particular groups corresponding to the category of moderate disease activity. After 1 year of prospective follow-up, we found a significant improvement in the status of patients achieving minimal disease activity, with the exception of patients treated with Golimumab. We found that patients in these groups had significantly lower disease activity than patients treated with (CZP) pegol (*p* = 0.015), Tocilizumab (*p* = 0.004) and Adalimumab (*p* = 0.003). Compared to other groups of biological therapy, there was no significant difference in disease activity.

**FIGURE 2:**
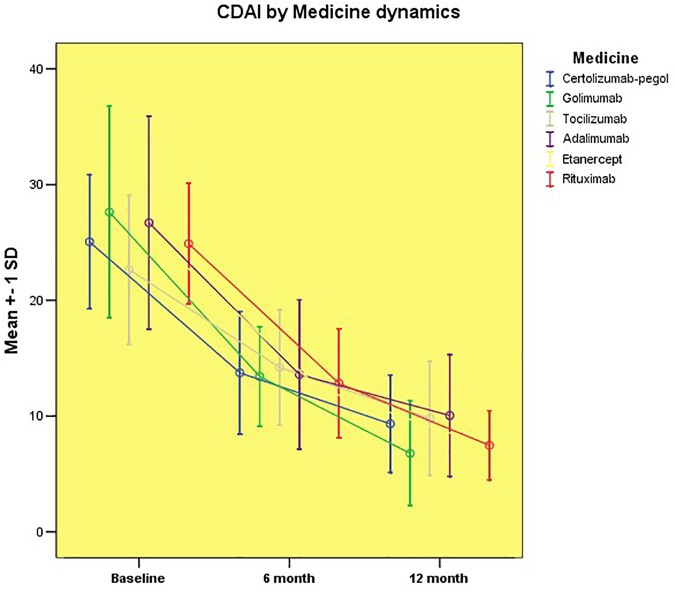
Disease activity assessed by CDAI by groups.

**FIGURE 3:**
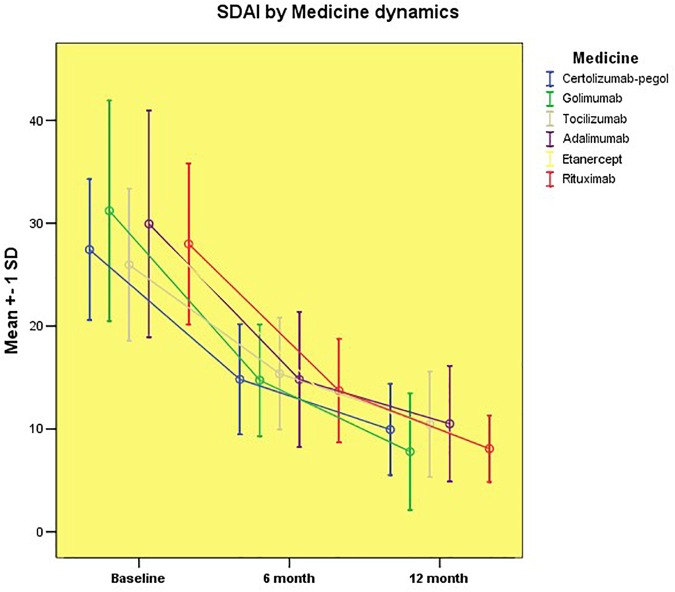
Disease activity assessed by SDAI by groups.

Similar results were obtained when evaluating disease activity by means of the clinical disease activity index (CDAI) and SDAI (Figures 2, 3).

An improvement was also observed for the 6 to 12 months of follow-up as we did not detect a significant difference in the activity of the disease assessed by CDAI among the different drug groups. In contrast to the results obtained for DAS28, after 1 year of follow-up, patients on Golimumab treatment did not achieve the remission category but a minimal disease activity.

The graphics depicting the change of the SDAI over the study period also outline comparable profiles. All of the treatment groups achieved a rapid reduction in disease activity that continued to decrease through the 6 and 12 months period, respectively, as supported by changes in SDAI. Patients treated with Golimumab and Rituximab achieved the lowest SDAI values (7.80 ± 5.67 and 8.08 ± 3.24, respectively) from all patient groups but still remained in the minimal disease activity category, and there was no statistically significant difference with the rest groups of biological treatment. After 1 year of follow-up, all groups reached the minimum disease activity category (Figure 3), demonstrating evidence of similar efficacy.

## Discussion

In clinical practice, there is a general agreement that rheumatoid inflammation should be controlled as soon as possible, as completely as possible, and that control should be maintained for as long as possible, consistent with patient safety (Wolfe et al., 2001). Following on from the aim of the treatment, the most important is to reach optimal control of rheumatoid inflammation or even remission. It is clear that the management of RA should include systematic and regular quantitative evaluation of rheumatoid inflammation and monitoring of long-term effects (Fransen et al., 2002). For the assessment of disease activity in daily clinical practice, DAS28, CDAI, and SDAI offer certain advantages (van Riel and van Gestel, 2001). The CDAI and SDAI are able to evaluate multiple parameters: swollen joint counts, tender joint counts, patient global assessment, physician global assessment and CRP (for SDAI) in a simple numerical summation (Smolen et al., 2003). Whereas swollen joint counts and physician global assessments are physician-derived assessments, tender joint counts (sometimes interpreted as a physician assessment) and patient global assessments depend upon the patient’s perception of disease activity. Thus the SDAI and CDAI give weight both to patient and physician assessments of disease activity (Boers et al., 1994; van Gestel et al., 1998). This parameter assessed by DAS28, CDAI and SDAI can be used as a guide in the suppression of RA disease activity with DMARDs or biologic DMARDS (Fransen et al., 2000).

The results from our study showed that treatment with biological agents significantly decreased the rheumatoid inflammation after a 12 month period of prospective treatment assessed by DAS28, CDAI and SDAI. All of the three tools show similar results for TNF inhibitors, except DAS28 for Golimumab after 12 months. The reason for this could be because of the small number of patients that were followed, because in general, it is clear that mean values of disease activity for each group are very close. The choice of biological treatment for RA may depend pragmatically on a number of factors, including patient preference, the tolerability of methotrexate and outpatient infusion facilities. Since a direct head-to-head comparison is not likely, it is difficult to determine if one biological drug works better than another in RA (Spofu et al., 2005). The Swedish Observational Study, for example, reported a lower drop-off rate and greater efficacy with regard to ACR 20 and 50 in patients taking etanercept compared with infliximab (Geborek et al., 2002). However, as with any observational study, there are many variables and it is important not to over-interpret this. Similarly, meta-analyses have suggested either that the three anti-TNF therapies are either equally effective in RA (Hochberg et al., 2003; Spofu et al., 2005) or that there are modest benefits in favor of etanercept compared with infliximab (Reynolds and Bacon, 2002) or adalimumab (Reynolds and Bacon, 2003). However, these studies have only been published so far only in abstract form.

In our study, we did not have the opportunity to include a group on therapy with Infliximab. The NHIF started to reimburse two biosimilars of Infliximab 3 years after the beginning of the study, which made it impossible for the prospective follow up in a 12 month period with a sufficient number of patients.

We have similar results from our study, which confirm these that were published in 2016 for the therapy with the Rituximab – ORBIT trial (Porter et al., 2016). The patients in our group on treatment with Rituximab significantly decreased their level of disease activity and did not have any significant differences of the level of rheumatoid inflammation with TNF inhibitors at the end of the trial period. This result of treatment with Rituximab is non-inferior to initial TNF inhibitor treatment in patients seropositive for RA.

According to the results for Tocilizumab in our study group, we can suggest that this biologic DMARD has similar results to TNF inhibitors. We confirm the results from the pan-European TOCERRA register collaboration, which shows that Tocilizumab has similar efficacy not only as a combined therapy with csDMARDS, but also as monotherapy in comparison with the TNF-inhibitors (Lauper et al., 2017).

This is the first national study evaluating three important disease activities markers in a follow up manner for 1 year period. It provides important information for the disease control and long-term effect of different bDMARD that could be used by rheumatologists in their everyday practice. Someone might consider the number of observed patients to be low but those are all patients on bDMARD therapy in the clinic and also significant part of all patients treated in the country. The NHIF rules are very restrictive in selecting biologic therapy. At the moment of observation only three clinics were allowed to prescribe biological therapy via specialized committees of three rheumatologists and our clinic is the biggest one. Additional limiting factor is also the consecutive appearance of the bDMARD on the national market and the long-term procedure for their inclusion in the reimbursement system.

These results could be used by health authorities to optimize RA therapy and better control the prescription of biologics. The usage of DAS28, SDAI and CDAI help the decision when it is the correct time to switch the patient onto another biologic therapy, due to insufficient or lack of clinical effect.

Our study had some limitations, such as the fact that the observation was done only in one clinic, although it is a national reference center for RA therapy. The second limitation was the small male sample size after recruitment. Further analysis will be done after new biosimilars or target synthetic DMARDS will be included in the reimbursement list of the NHIF.

## Conclusion

Therapy with all of the bDMARDS significantly decrease the rheumatoid inflammation measured by DAS28, CDAI and SDAI. All of the biological agents achieved the target for the treatment of RA – a minimal disease activity level, and they appear to be similarly effective.

## Ethics Statement

The ethics committee at the University Hospital “St. Ivan Rilski” in Sofia approved the study. The study is in accordance with the requirements of Helsinki Declaration. Only capable and willing to participate patients was recruited. It is an observational, prospective, real life study.

## Author Contributions

VB, NS, and RS collected the data, and analyzed and interpreted the results. MI participated in the statistical evaluation and data analysis. RS and GP designed the study, monitored its progress, and revised the article text. All authors revised and accepted the manuscript.

## Conflict of Interest Statement

The authors declare that the research was conducted in the absence of any commercial or financial relationships that could be construed as a potential conflict of interest.
